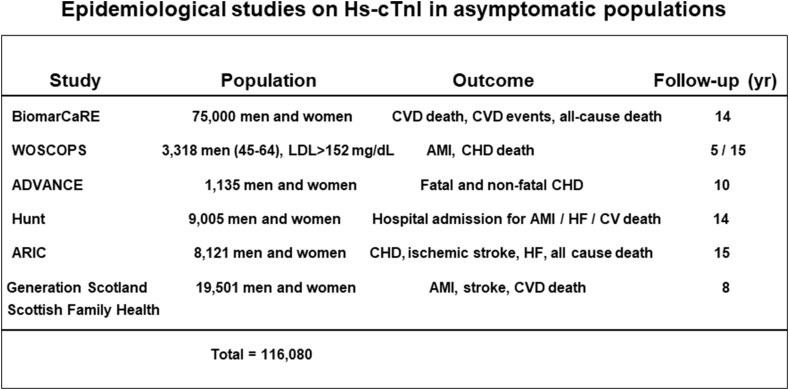# High-sensitivity troponin I is associated with cardiovascular outcomes but not with breast arterial calcification among postmenopausal women

**DOI:** 10.1016/j.ijcrp.2022.200157

**Published:** 2022-11-01

**Authors:** Carlos Iribarren, Malini Chandra, Catherine Lee, Gabriela Sanchez, Danny L. Sam, Farima Faith Azamian, Hyo-Min Cho, Huanjun Ding, Nathan D. Wong, Sabee Molloi

**Affiliations:** a Kaiser Permanente Division of Research, 2000 Broadway, Oakland, CA, USA; b Kaiser Permanente Santa Clara Medical Center, Santa Clara, CA, USA; c Department of Radiological Sciences, University of California Irvine School of Medicine, Irvine, CA, USA; d Medical Measurement Team, Korea Research Institute of Standards and Science, Daejeon, South Korea; e Division of Cardiology, Department of Medicine and Department of Epidemiology, University of California Irvine, Irvine, CA, USA

**Keywords:** High-sensitivity troponin I, Breast arterial calcification, Cardiovascular disease, Women's health, Cohort study

## Abstract

**Background:**

Prior studies support the utility of high sensitivity troponin I (hsTnI) for cardiovascular disease (CVD) risk stratification among asymptomatic populations; however, only two prior studies examined women separately. The association between hsTnI and breast arterial calcification is unknown.

**Methods:**

Cohort study of 2896 women aged 60–79 years recruited after attending mammography screening between 10/2012 and 2/2015. BAC status (presence versus absence) and quantity (calcium mass mg) was determined using digital mammograms. Pre-specified endpoints were incident coronary heart disease (CHD), ischemic stroke, heart failure and its subtypes and all CVD.

**Results:**

After 7.4 (SD = 1.7) years of follow-up, 51 CHD, 30 ischemic stroke and 46 heart failure events were ascertained. At a limit of detection of 1.6 ng/L, 98.3 of the cohort had measurable hsTnI concentration. HsTnI in the 4–10 ng/L range were independently associated of CHD (adjusted hazard ratio[aHR] = 2.78; 95% CI, 1.48–5.22; p = 0.002) and all CVD (aHR = 2.06; 95% CI, 1.37–3.09; p = 0.0005) and hsTnI over 10 ng/L was independently associated with CHD (aHR = 4.75; 95% CI, 1.83–12.3; p = 0.001), ischemic stroke (aHR = 3.81; 95% CI, 1.22–11.9; p = 0.02), heart failure (aHR = 3.29; 95% CI, 1.33–8.13; p = 0.01) and all CVD (aHR = 4.78; 95% CI, 2.66–8.59; p < 0.0001). No significant association was found between hsTnI and BAC. Adding hsTnI to a model containing the Pooled Cohorts Equation resulted in significant and clinical important improved calibration, discrimination (Δ C_index_ = 6.5; p = 0.02) and reclassification (bias-corrected clinical NRI = 0.18; 95% CI, −0.13-0.49 after adding hsTnI categories).

**Conclusions:**

Our results support the consideration of hsTnI as a risk enhancing factor for CVD in asymptomatic women that could drive preventive or therapeutic decisions.

## Non-standard abbreviations

BAC: Breast arterial calcification
CAC: Coronary artery calcification
CAD: Coronary artery disease
CHD: Coronary heart disease
CVD: Cardiovascular disease
ASCVD: Atherosclerotic cardiovascular disease
CI: Confidence Interval
PCE: American Heart Association/American College of Cardiology Pooled Cohorts Equation
NRI: Net reclassification improvement
cNRI: Clinical net reclassification improvement
MINERVA: **M**ult**I**eth**N**ic study of br**E**ast a**R**terial calcium gradation and cardio**VA**scular disease
KPNC: Kaiser Permanente of Northern California
MLO: Mediolateral oblique
CC: Craniocaudal
ICD-9: International classification of diseases 9th revision
ICD-10: International classification of diseases 10th revision
CPT4: Current procedure terminology, 4th edition
PPV: Positive predictive value
SD: Standard deviation
SBP: Systolic blood pressure
DBP: Diastolic blood pressure
LDL: Low density lipoprotein
HDL: High density lipoprotein
BMI: Body mass index
HsTnI: High sensitivity troponin I
Hs-CRP: High-sensitive C-reactive protein
BNP: Brain natriuretic peptide
EF: Ejection fraction
HFpEF: Heart failure with preserved ejection fraction
HFrEF: Heart failure with reduced ejection fraction

## 1 Background

Troponin I and T are protein complexes that regulate muscle contraction [1]. Whereas troponin I is specific to heart myocytes, troponin T can derive from skeletal muscle as well [2]. Therefore, cardiac troponin I is the preferred biomarker for the evaluation of myocardial injury and diagnosis of acute myocardial infarction [3]. A new generation of high-sensitivity cardiac troponin assays are recommended for routine clinical use [1]. Besides its use in the acute setting, a body of epidemiological research [[4], [5], [6], [7], [8], [9]] supports the utility of hsTnI for cardiovascular risk stratification among asymptomatic populations. However, only two of those studies examined women separately [6,7].

Breast arterial calcification (BAC) is commonly seen in routine screening mammograms and is currently not considered a clinically-actionable incidental finding. However, many studies have shown that BAC is associated with angiographically-defined CAD [[10], [11], [12], [13]] and with increased risk of CVD outcomes [[14], [15], [16], [17], [18]]. The pathophysiology of BAC is not completely understood. Studies have shown consistent relationships of BAC with age, hypertension and diabetes, but no relationships with smoking or hyperlipidemia [19]. The association of BAC with hsTnI has not been investigated.

The aims of this study were two-fold. First, to further validate the utility of hsTnI for CVD risk stratification in postmenopausal women; and second, to shed light on the pathophysiology of BAC by examining the association between BAC and hsTnI.

## 2 Methods

The data that support the findings of this study are available from the corresponding author upon reasonable request.

### 2.1 Cohort description

**Fig. 1:**
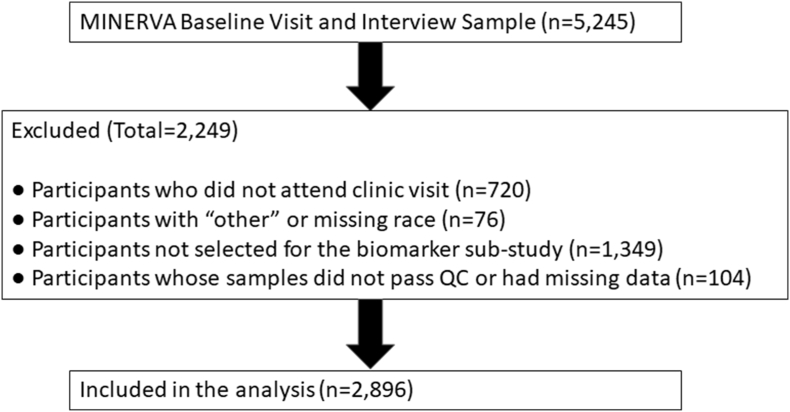
Flow diagram of cohort selection.

MINERVA (**M**ult**I**eth**N**ic study of br**E**ast a**R**terial calcium gradation and cardio**VA**scular disease) is a large, racially and ethnically diverse cohort of postmenopausal women. Details of recruitment, study procedures and baseline characteristics are published elsewhere [20]. In brief, eligible participants were female active members of Kaiser Permanente of Northern California (KPNC) between the ages of 60 and 79 when they attended regular mammography screening at KPNC between 10/24/2012 and 2/13/2015. Women attending mammography for diagnostic purposes were not eligible. Those with a prior history of myocardial infarction, coronary revascularization, stroke, heart failure, peripheral vascular disease, breast cancer, mastectomy or breast implants, Alzheimer's disease/dementia, chronic dialysis/renal transplant or not having an assigned primary care provider were also excluded. A total of 201,830 women underwent screening mammography at the study centers, and 46,112 met eligibility criteria. Of those, 4425 women attended clinic visits and had available stored blood samples, and 720 consented to participate, responded to an abbreviated questionnaire and did not attend the clinic visit or provided blood samples. For budgetary reasons, 3000 women who attended the clinic visits and had available stored blood samples were randomly selected for the MINERVA Biomarkers sub-study that the current study is based on. The final sample for analysis after quality control procedures and excluding missing covariates was 2896. Fig. 1 details the consort diagram of cohort selection. Compared with the complete cohort of 5145 women, the women in this sub-study were slightly older (66.4 vs 65.7 years), more likely to be white (69 vs. 52%) and had a greater prevalence of BAC (32 vs 26%). The study was approved by the Institutional Review Boards of the participating institutions and all participants signed an informed consent.

### 2.2 Study procedures

#### 2.2.1 BAC assessment

All images were acquired using full-field digital mammography units (Senographe 2000D, General Electric Medical Systems, Milwaukee, WI or Selenia Hologic, Hologic Inc., Malborough, MA). Standard full-field digital mammograms were acquired from mediolateral oblique (MLO) and craniocaudal (CC) projections. A new validated densitometry method was used to estimate a continuous BAC mass (in milligrams [mg]) score using raw (uncompressed) digital mammograms prospectively acquired and transmitted to the BAC Reading Center at the Department of Radiological Sciences, University of California Irvine School of Medicine [21]. Intra- and inter-machine variability has been addressed before [22].

### 2.3 Cardiovascular outcomes

Incident coronary heart disease (including acute myocardial infarction, coronary revascularization procedures and CHD death), ischemic stroke and heart failure were ascertained through September 30, 2021 using standard validated inpatient ICD-9, ICD-10 and CPT4 procedure codes, plus underlying cause of death (see STable 1). To determine the heart failure type, a physician investigator (C·I.) reviewed the medical records of the 46 women found to have a diagnosis of heart failure to extract ejection fraction (EF) data from the echocardiogram closest to the event. Twenty-nine (63%) had EF ≥ 50% and were classified as heart failure with preserved ejection fraction (HFpEF), and 17 (37%) had EF < 50% and were classified as heart failure with reduced EF (HFrEF). The validity of the event ascertainment using codes and underlying cause of death has been reported elsewhere [23]. The mean (SD) length of follow-up was 7.4 (1.7 years).

### 2.4 Covariate assessment

Age, race/ethnicity and smoking status were ascertained with a self-administered questionnaire. Clinic visits took place, on average, 3.2 months (SD = 3.0) after the screening mammography. Details of clinic procedures and laboratory methods can be found elsewhere [20]. Body mass index was estimated as weight in kg over height in m^2^. Glycemic status was defined as normoglycemia, prediabetes and diabetes diagnosis or treatment. Hypertension was defined as self-report of hypertension and/or self-report of treatment for hypertension and/or systolic blood pressure (SBP) ≥ 140 mmHg and/or diastolic blood pressure (DBP) ≥ 90 mmHg.

### 2.5 Biomarkers

High-sensitive C-reactive protein (hs- CRP) was measured using a chemiluminescent assay (Siemens Healthcare Diagnostics, Deerfield, IL) with a coefficient of variation of 6%. HsTnI concentrations were measured on serum specimens using the ARCHITECT STAT High Sensitive Troponin I assay (Abbott Laboratories, Abbott Park, IL) [24]. This assay is designed to have within-laboratory CV of ≤10% at a concentration of 5.2 pg/mL and to have analytical specificity of ≤0.1% cross reactivity with skeletal troponin-I and ≤1% cross reactivity with cardiac troponin T and troponin C. The lower limit of detection range of this assay is 0.7–1.6 ng/L [24]. Brain natriuretic peptide (BNP) was measured with the ARCHITECT BNP Assay, a chemiluminescent microparticle immunoassay (CMIA) which has a total CV between 1.7 and 6.7%.

### 2.6 Statistical methods

We first described the analytical cohort using means and standard deviations of continuous variables and frequency distributions of categorical variables. For variables that were not normally-distributed (hs-CRP, hsTnI and BNP) were report median and interquartile range. Differences in continuous variables across hsTnI levels (<4, 4–10 and > 10 ng/L) were assessed with analysis of variance (ANOVA) for normally distributed continuous variables, with the Kruskal-Wallis test for not-normally distributed continuous variables, and with the Chi-square test for categorical variables. Age-adjusted rates per 1000 person-years of CHD, ischemic stroke, heart failure (and heart failure types) and all CVD were estimated according to hsTnI levels using Poisson regression with right-censoring at death or termination of health plan membership (n = 441; 15.2%). We used the Kaplan-Meier method to compare survival curves across hs TnI groups for all outcomes. Hazard ratios and 95% confidence intervals associated with hsTnI between 4 and 10 ng/dL and >10 ng/L compared with hsTnI <4 ng/dL were estimated using Cox proportional hazards models with minimal adjustment (Model 1: age, race/ethnicity) and full adjustment for age, race/ethnicity, smoking status, BMI, hypertension, glycemic status, total cholesterol/HDL ratio, cholesterol lowering drugs and hs-CRP (Model 2). In heart failure models, BNP was also entered as a covariate in Model 2. We also ran complementary models for all outcomes where hsTnI was parametrized as a continuous standardized effect (i.e., one standard deviation increment). We examined whether risk estimates derived from models using the Pooled Cohorts Equation (PCE) risk and hsTnI have better performance than risk estimates derived from models using PCE risk solely. We estimated 5-year cumulative incidence of hard atherosclerotic cardiovascular disease (ASCVD, which comprised CHD and ischemic stroke) using a Cox model that included PCE and hsTnI, where hsTnI was a three-level categorical variable (<4, 4–10 and > 10 ng/L) and compared these risks to those estimated using PCE alone. We examined calibration visually through calibration plots and analytically using the Greenwood-D’Agostino-Nam test for calibration [25,26]. We assessed discrimination using the Harrell C-index [27]. We then estimated the overall category-based net reclassification index (NRI) to assess the extent to which adding hsTnI to the risk models moves risk upward among ASCVD cases and downward among non-cases, and the bias-corrected clinical NRI (cNRI) among those with borderline or intermediate risk using the standard PCE categories of <5, 5 to <7.5, 7.5 to 20 and ≥ 20%, respectively [28,29]. The association between hsTnI and BAC was examined first in a bivariate fashion by estimating proportions with BAC in each hsTnI category and applying the Chi-square test. This was done for varying BAC thresholds including 0, 5, 10 and 25 mg. The independent association of hsTnI with BAC (also at different thresholds) was assessed using logistic regression with minimal and full adjustment as implemented in the CVD outcome analysis. Statistical analyses were performed with SAS version 9.4 and R version 4.0.5. Significance level was set at p < 0.05.

## 3 Results

The analytical cohort was, on average (SD), 66.4 (4.4) years old and 70% white, 10% black, 9.5% Hispanic/Latina, and 11% Asian (Table 1). Only 3.5 were current smokers and 38% former smokers. Whereas 43% were in the pre-diabetic range, 13% had diabetes. Fifty-six % had hypertension and 76% were on cholesterol lowering medication. At a limit of detection of 1.6 ng/L, 98.3% of the sample had measurable levels of hsTnI. The median hsTnI was 3.6 ng/L and the interquartile range was 2.8 (25th percentile) to 4.7 (75th percentile) ng/L. The distribution of hsTnI levels were as follows: 59% < 4 ng/L, 36% 4–10 ng/L and 5% > 10 ng/L. Any BAC was present in 32%, BAC >5 mg in 13%, BAC >10 mg in 9% and BAC >25 mg in 4% (Table 1). Hs TnI was positively associated with age, African-American and Asian race/ethnicity, BMI, hs CRP and BNP (Table 1). After a mean (SD) follow-up of 7.4 (1.7) years, 51 CHD, 30 ischemic stroke and 46 heart failure events were ascertained. As shown in Table 2, there was a stepwise increase in age-adjusted rates per 1000 person-years of all CVD events with increasing concentration of hsTnI (p-trend≤0.03). The Kaplan-Meier survival curves shows clear separation of hsTnI groups, more clearly for any CVD and heart failure, but statistically significant (p < 0.04) for all outcomes (SFig. 1). In Cox regression with full adjustment, hsTnI between 4 and 10 ng/L (relative to < 4 ng/dL) was associated with 2.8-fold significant increased hazard of CHD (p = 0.002) and 2.0-fold significant increased hazard of all CVD (p = 0.0005) (Table 3). Also in fully adjusted models, hsTnI >10 ng/L (relative to < 4 ng/dL) was associated with 4.7-fold significant increased hazard of CHD (p = 0.001), 3.8-fold significant increased hazard of ischemic stroke (p = 0.02), 3.3-fold significant increased hazard of heart failure (p = 0.01) and 4.8-fold significant increased hazard of any CVD (p < 0.0001) (Table 3). When hsTnI was entered as a standardized continuous variable, the fully-adjusted hazard ratio varied between 1.28 (95% CI, 1.09–1.52; p = 0.003) for heart failure and 1.31(95% CI, 1.15–1.50; p < 0.0001) for CHD. When the association of hsTnI with heart failure was examined by heart failure types, we found that it was driven by HFrEF (STable 2 and STable 3). In the fully-adjusted model, hsTnI >10 ng/L (relative to < 4 ng/dL) was associated with 25-fold significant increased hazard of HFrEF (p = 0.0001). One S.D. increment in hsTnI was associated with 1.59 (95% CI, 1.32–1.90; p < 0.0001) increased hazard of HFrEF and no significant association was seen for HFpEF (HR = 0.93; 95% CI, 0.63–1.37; p = 0.70).

**Table 1:** MINERVA biomarkers cohort baseline characteristics (n = 2896).

	High-sensitivity cardiac Troponin I (ng/L)				
	<4 n = 1715 (59.2%)	4-10 n = 1039 (35.9%)	>10 n = 142 (4.9%)	p-value^a^	All N = 2896 (100%)
**Demographics**					
Age (years), mean ± SD	66.1 ± 4.3	66.9 ± 4.6	66.8 ± 4.5	<.0001	66.4 ± 4.4
Race/Ethnicity, n (%)				0.0001	
White	1221 (71.2%)	708 (68.1%)	91 (64.1%)		2020 (69.8%)
Black	135 (7.9%)	139 (13.4%)	19 (13.4%)		293 (10.1%)
Hispanic/Latina	178 (10.4%)	84 (8.1%)	13 (9.2%)		275 (9.5%)
Asian	181 (10.6%)	108 (10.4%)	19 (13.4%)		308 (10.6%)
**Lifestyle and Clinical Variables**					
Smoking status, n (%)				0.99	
Never	1011 (59.0%)	609 (58.6%)	84 (59.2%)		1704 (58.8%)
Former	642 (37.4%)	394 (37.9%)	54 (38.0%)		1090 (37.6%)
Current	62 (3.6%)	36 (3.5%)	4 (2.8%)		102 (3.5%)
BMI (Kg/m2), mean ± SD	27.3 ± 5.6	28.3 ± 6.9	28.5 ± 7.0	<.0001	27.7 ± 6.1
Total cholesterol (mg/dL), mean ± S	208 ± 36	210 ± 38	205 ± 39	0.13	208 ± 37
LDL-C (mg/dL), mean ± SD	121 ± 31	123 ± 34	119 ± 35	0.8	122 ± 32
HDL-C (mg/dL), mean ± SD	67 ± 17	66 ± 17	66 ± 17	0.77	66 ± 17
Hs CRP (mg/dL), median (q1-q3)	1.4 (0.7–3.5)	1.6 (0.7–4.2)	1.8 (0.7–4.0)	0.03	1.5 (0.7–3.8)
Glycemic status, n (%)				0.10	
Normoglycemia	789 (46.0%)	428 (41.2%)	57 (40.1%)		1274 (44.0%)
Prediabetes	716 (41.8%)	465 (44.8%)	68 (47.9%)		1249 (43.1%)
Diabetes diagnosis or treatment	210 (12.2%)	146 (14.1%)	17 (12.0%)		373 (12.9%)
Hypertension, n (%)				<0.0001	
No	843 (49.2%)	376 (36.2%)	48 (33.8%)		1267 (43.8%)
Yes	872 (50.9%)	663 (63.8%)	94 (66.2%)		1629 (56.3%)
On cholesterol lowering drugs, n (%)				0.41	
No	407 (23.7%)	261 (25.1%)	29 (20.4%)		697 (24.1%)
Yes	1308 (76.3%)	778 (74.9%)	113 (79.6%)		2199 (75.9%)
**High-sensitivity cardiac Troponin I (ng/L)**					
Median (q1-q3)	3.0 (2.5–3.4)	4.9 (4.4–5.9)	15.5 (11.7–25.0)	<.0001	3.6 (2.8–4.7)
**Brain natriuretic peptide (BNP) (ng/L)**					
Median (q1-q3)	19.3 (11.4–31.9)	21.7 (12.5–40.9)	25.6 (14.4–43.1)	<.0001	20.4 (11.9–35.7)
**BAC prevalence, n (%)**					
BAC >0 mg	543 (31.7%)	346 (33.3%)	52 (36.6%)	0.38	941 (32.5%)
BAC >5 mg	214 (12.5%)	135 (13.0%)	21 (14.8%)	0.71	370 (12.8%)
BAC >10 mg	141 (8.2%)	97 (9.3%)	14 (9.9%)	0.53	252 (8.7%)
BAC >25 mg	56 (3.3%)	46 (4.4%)	6 (4.2%)	0.28	108 (3.7%)

a p-ANOVA or Chi-square test.

**Table 2:** CVD Incidence (Age-adjusted Rates per 1000 Person-years and SE) by hsTnI levels (n = 2896).

hsTnI (ng/L)	Coronary Heart Disease (n = 51)	Ischemic Stroke (n = 30)	Heart Failure (n = 46)	All (n = 116)
<4	n = 15	n = 13	n = 16	n = 41
n = 1715 (59.2%)	1.1 ± 2.0	1.0 ± 2.8	1.2 ± 2.1	3.2 ± 1.3
4–10	n = 30	n = 13	n = 21	n = 59
n = 1039 (35.8%)	3.5 ± 2.0	1.7 ± 2.8	2.3 ± 2.1	7.5 ± 1.4
>10	n = 6	n = 4	n = 9	n = 16
n = 142 (4.9%)	5.2 ± 2.1	3.9 ± 2.9	7.6 ± 2.1	15.6 ± 1.4
p-trend	0.0003	0.03	0.0003	<.0001

**Table 3:** Independent Associations of hsTnI with CVD events (n = 2896).

hsTnI (ng/L)	Coronary Heart Disease (n = 51)	Ischemic Stroke (n = 30)	Heart Failure (n = 46)	All (n = 116)
**Hazard Ratios (95% CI); p-value**				
<4 (n = 1715; 59.2%)				
Model 1	1.00	1.00	1.00	1.00
Model 2	1.00	1.00	1.00	1.00
4–10 (n = 1039; 35.8%)				
Model 1	3.21 (1.72–5.98); 0.0003	1.66 (0.77–3.61); 0.20	1.98 (1.03–3.80); 0.04	2.35 (1.57–3.51); <.0001
Model 2	2.78 (1.48–5.22); 0.002	1.55 (0.71–3.42); 0.27	1.39 (0.70–2.73); 0.35	2.06 (1.37–3.09); 0.0005
>10 (n = 142; 4.9%)				
Model 1	4.87 (1.88–12.57); 0.001	3.82 (1.24–11.75); 0.02	6.54 (2.88–14.84); <.0001	4.95 (2.77–8.83); <.0001
Model 2	4.75 (1.83–12.32); 0.001	3.81 (1.22–11.86); 0.02	3.29 (1.33–8.13); 0.01	4.78 (2.66–8.59); <.0001
1 SD increment in log (hsTnI)				
Model 1	1.32 (1.16–1.49); <.0001	1.30 (1.07–1.58); 0.01	1.36 (1.20–1.53); <.0001	1.32 (1.21–1.44); <.0001
Model 2	1.31 (1.15–1.50); <.0001	1.29 (1.05–1.58); 0.02	1.28 (1.09–1.52); 0.003	1.30 (1.19–1.43); <.0001

Model 1: age- and race/ethnicity-adjusted.

Model 2: adjusted for age, race/ethnicity, smoking, BMI, hypertension, glycemic status, total cholesterol/HDL ratio, cholesterol lowering drugs, hs-CRP.

Model 2 for heart failure adjusts also for BNP.

There were no significant trends of increased BAC prevalence at any BAC threshold with increasing level of hsTnI (p ≥ 0.28) (Table 4). Likewise, hsTnI 4–10 ng/L or >10 ng/L or hsTnI as a continuous variable were not statistically significantly associated with BAC at any BAC threshold level (p ≥ 0.33) (STable 4).

**Table 4:** Prevalence of BAC by hsTnI levels (n = 2896).

hsTnI (ng/L)	BAC >0 mg	BAC >5 mg	BAC >10 mg	BAC >25 mg
	N = 941	N = 370	N = 252	N = 108
<4	543 (31.7%)	214 (12.5%)	141 (8.2%)	56 (3.3%)
n = 1715 (59.2%)				
4–10	346 (33.3%)	135 (13.0%)	97 (9.3%)	46 (4.4%)
n = 1039 (35.8%)				
>10	52 (36.6%)	21 (14.8%)	14 (9.9%)	6 (4.2%)
n = 142 (4.9%)				
Chi Square p-value*	0.38	0.71	0.53	0.28

Addition of hsTnI categories to a model already containing the PCE resulted in a well-calibrated model (p = 0.96) (STable 5). The C-index, a measure of discrimination, for the model containing only PCE risk was 61.7 and it significantly increased (Δ C-index = 6.5; p = 0.02) to 68.2 after adding hs-cTnI categories. The overall category-based net reclassification improvement (NRI) was 0.32 (95% CI, 0.07–0.58) and the bias-corrected clinical NRI was 0.18 (95% CI, −0.13-0.49) after adding hsTnI categories. The reclassification tables are provided in STable 6. The gains in reclassification were mostly driven by up-risking of women with high levels of hsTnI.

## 4 Discussion

Our main findings in a large cohort of women who were 60–79 years old at baseline are that hsTnI in the 4–10 ng/L range were independently associated of CHD and all CVD and hsTnI over 10 ng/L and hsTnI as a continuous variable were independently associated with CHD, ischemic stroke, heart failure and all CVD. We found that adding hsTnI to a ASCVD model containing the PCE resulted in significant and clinical important improved calibration, discrimination and reclassification, supporting the utility of hsTnI as a biomarker for CVD risk stratification in asymptomatic postmenopausal women. It is unlikely in our cohort that the elevated hsTnI levels were secondary to prior CVD or end-stage renal disease because these were exclusionary conditions [30]. Furthermore, it is also very unlikely that study participants had other potential secondary causes of hsTnI elevation such as a major fall or accident, major surgery, sepsis, cardiotoxic chemotherapy or an episode of strenuous exercise just before the clinic visit [31]. Therefore, the most probable scenario is that the elevated hsTnI truly represents subclinical myocardial injury.

Several prior cohort studies in asymptomatic populations have consistently reported significant independent associations of hsTnI with CVD outcomes [[4], [5], [6], [7], [8]], but most of them presented results of men and women combined (see SFig. 2). The West of Scotland Coronary Prevention Study (WOSCOPS) among men 45–64 years old with LDL-C > 152 mg/dL concluded that troponin concentration predicted coronary events, was reduced by statin therapy, and change at 1 year was associated with future coronary risk independent of cholesterol lowering [9]. It is not straightforward to compare results across studies because investigators used different endpoints, and the age, composition of the samples and follow-up time also differed across studies. The two studies that reported sex-specific results were ARIC(6) and the Generation Scotland Scottish Family Health Study (GSSFHS) [7], and in both instances a stronger association of hsTnI with CHD in ARIC and a composite outcome of cardiovascular death, myocardial infarction or stroke in GSSFHS were noted in women compared with men. MINERVA is therefore the first women-only cohort study examining the link between hsTnI and CVD in the primary prevention setting.

Our analysis failed to detect an association between hsTnI levels and BAC, suggesting that subclinical myocardial ischemia is not likely to be etiologically linked to BAC. A Danish study reported that hsTnI was able to predict presence of coronary artery calcification (CAC), with an odds ratio for an Agatston score >0 of 1.25 (95% CI: 1.03–1.51; p = 0.025) and 1.36 (95% CI: 1.08–1.71; p = 0.009) for an Agatston score >100 [32]. BAC and CAC are two vascular calcification phenotypes with different pathophysiology and etiology: whereas CAC represents intimal calcium deposits related to the atherosclerotic process (and is closely related to smoking and hyperlipidemia), BAC represents medial calcium deposits leading to arterial stiffness and is related more closely to diabetes and hypertension [[33], [34], [35]]. The arterial supply to the breast is primarily derived from branches of the internal thoracic (mammary) artery, intercostal arteries, and the lateral thoracic artery. Arterial branches of the internal and lateral thoracic arteries branch out deep into the breast parenchyma. BAC is almost exclusive for arteries, as venous calcifications in the breast are rare. Because of size (small to medium) and biology, breast arteries are comparable to epicardial or microvascular circulation and distinct from large vessels such as the coronaries or the aorta.

Strengths of the MINERVA cohort include: the large size, deep rigorous phenotyping of risk factors and lifestyle, ethnic diversity, availability of 7.4 years of follow up, and objective quantitative assessment of BAC quantity using contemporary digital mammography. We recognize several limitations. First, our findings may not be generalizable to uninsured populations or to women younger than 60. Second, the densitometry method for assessment of BAC quantity has only been validated by our group, so external validation is warranted. Finally, we did not assess the number of calcified vascular lesions in the breast, thus we were unable to examine the importance of localized versus diffuse BAC.

In conclusion, our analysis in a large, ethnically diverse cohort of postmenopausal demonstrates that hsTnI is independently related to incident CVD and its components, CHD and ischemic stroke as well as heart failure, particularly HFrEF. Cardiac troponin is not currently part of the standard for risk stratification of asymptomatic women. Our results, together with the accumulated evidence over the last few years supports the consideration of hsTnI as a risk enhancing factor for CVD in women that could drive preventive or therapeutic decisions.

## Funding

MINERVA is an investigator-initiated cohort study funded by the 10.13039/100000050National Heart, Lung, and Blood Institute (RO1HL106-043; multiple PIs Iribarren and Molloi).

## Declaration of competing interest

C.I. is a consultant for Abbott Diagnostics. 10.13039/100014386Abbott Diagnostics performed the ARCHITECT assays for hs-cTn I and BNP and provided financial support to the MINERVA Biomarkers Study.

## Figures and Tables

**figs1:**