# Supplementary figures and images for: High-sensitivity troponin I is associated with cardiovascular outcomes but not with breast arterial calcification among postmenopausal women
Source: Int J Cardiol Cardiovasc Risk Prev. 2022 Nov 1;15:200157. doi: 10.1016/j.ijcrp.2022.200157 (PMC9789357; doi:10.1016/j.ijcrp.2022.200157)

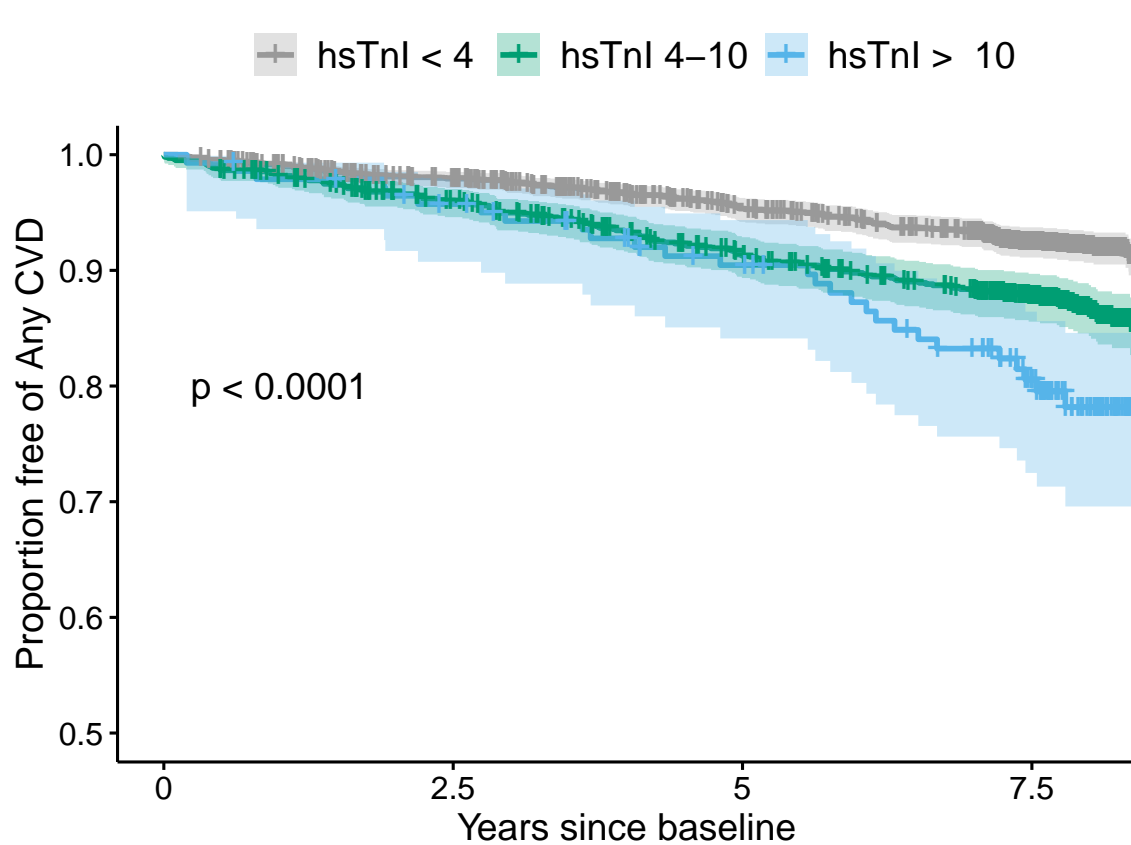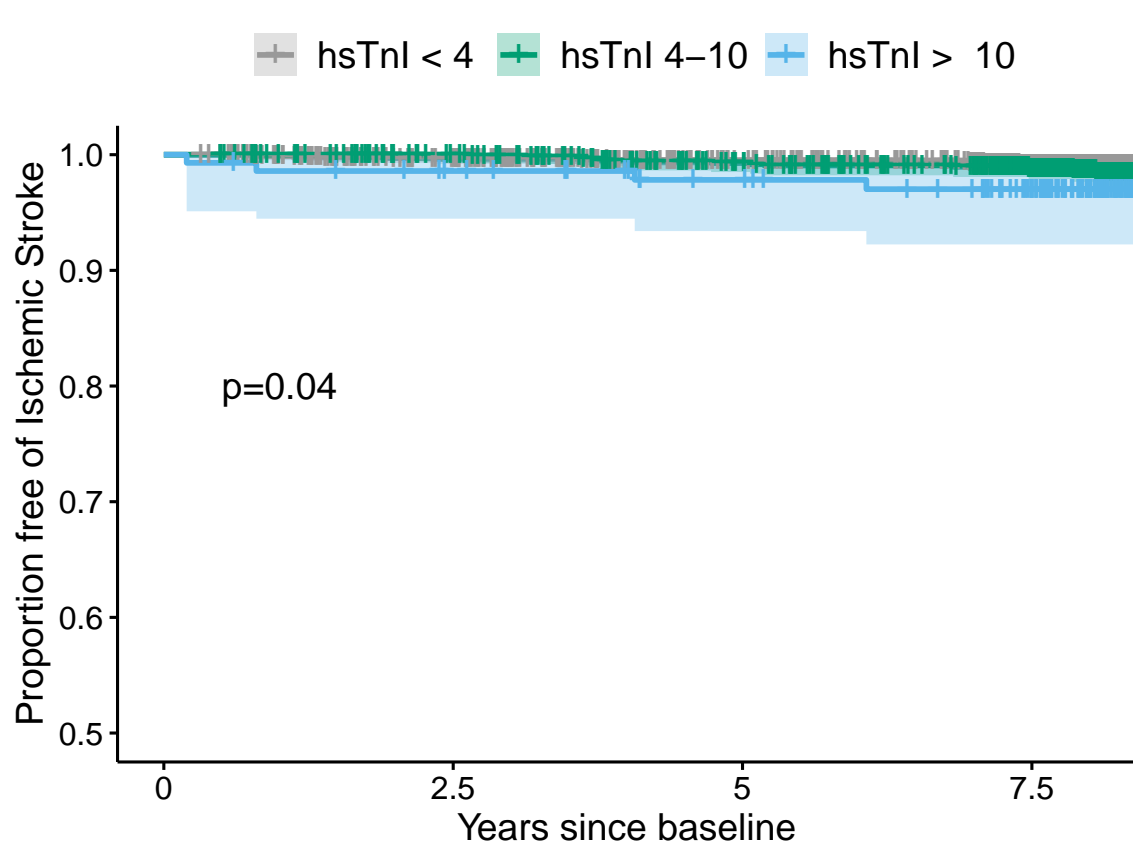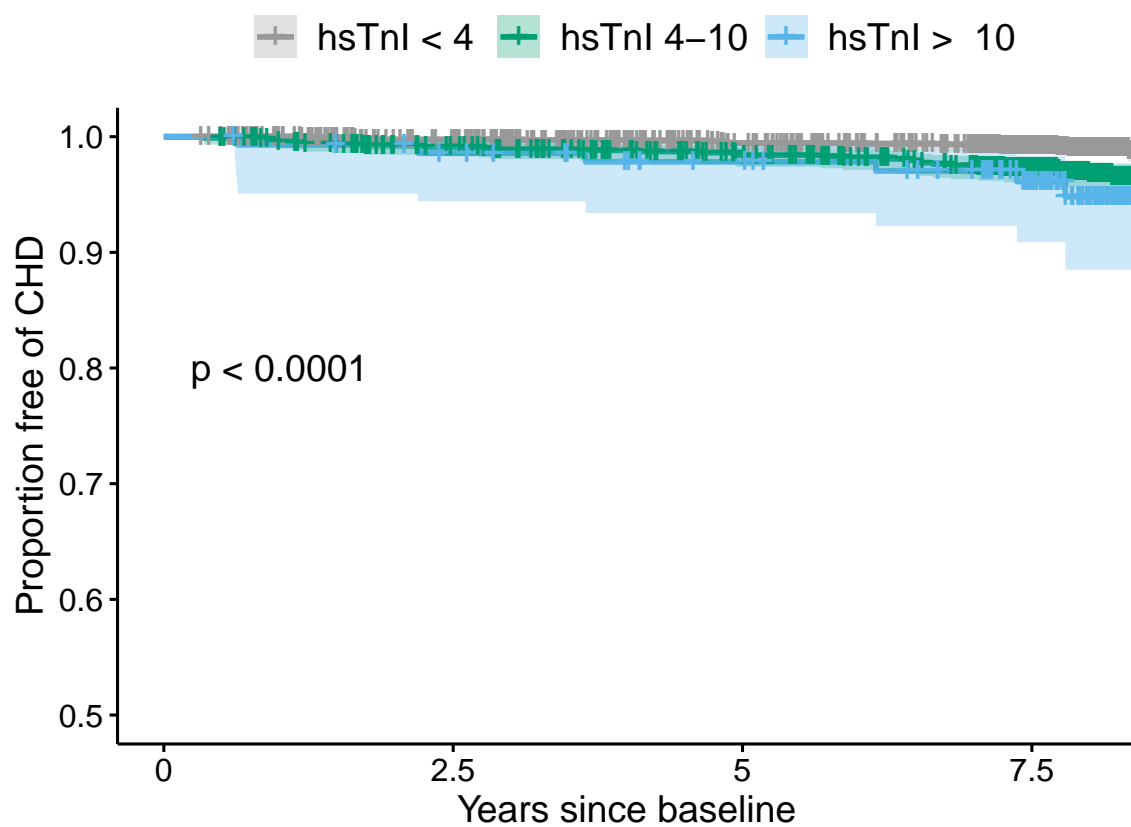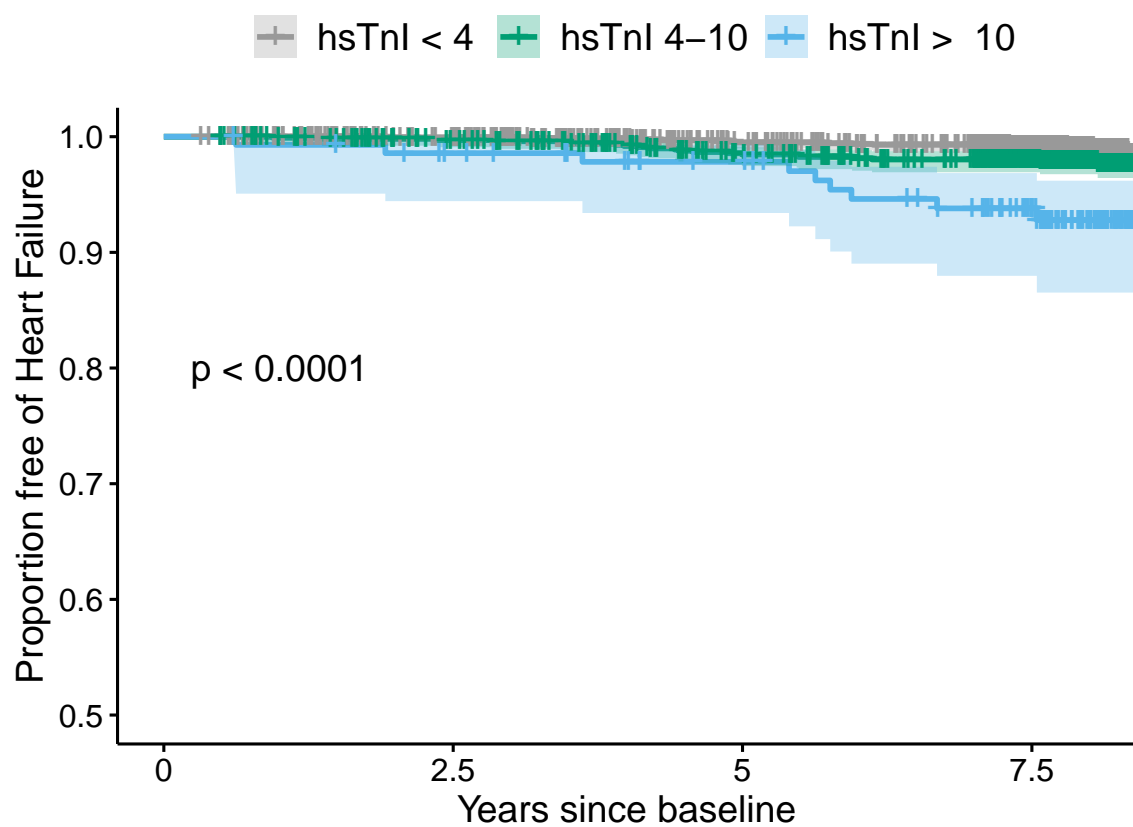

Supplement: Multimedia component 1 [file mmc1.pdf]
