# Supplementary material for: High-sensitivity troponin I is associated with cardiovascular outcomes but not with breast arterial calcification among postmenopausal women
Source: Int J Cardiol Cardiovasc Risk Prev. 2022 Nov 1;15:200157. doi: 10.1016/j.ijcrp.2022.200157 (PMC9789357; doi:10.1016/j.ijcrp.2022.200157)
Supplement: Multimedia component 2 [file mmc2.docx]

**Table S1.** Codes for CVD Outcome Ascertainment.

|  | ICD-9 | ICD-10 | CPT4 Procedure Codes |
| --- | --- | --- | --- |
| Coronary Heart Disease |  |  |  |
| Acute myocardial infarction | 410, 412 | I21, I22, I25.6 |  |
| Coronary angioplasty/stent/bypass graft surgery | 36.01, 36.02,36.03,36.05, 36.06, 36.07, 36.09, 36.10, 36.11, 36.12, 36.13, 36.14, 36.15, 36.16, 36.17, 36.19, 36.03, 36.2, 36.3, 00.66, 295.5, V45.81, V45.82 | ICD-10-PCS: 0210*, 0211*, 0212*, 0213*, 2703*, 02713*, 02723*, 02733*, Z95.5, Z98.61 | 92980, 92981, 92982, 92984, 92995, 92996, 92975, 92977, 33510, 33511, 92920, 92921, 92924, 92925, 92928, 92929, 92933, 92934, 92937, 92938, 92941, 92943, 92944, 33510-33536 |
| Ischemic stroke | 433.01, 433.11, 433.21, 433.31, 433.81, 433.91, 434.01, 434.11, 434.91, 437.0, 437.1 | I63, I67.89, I69.320, I69.398 |  |
| Heart failure | 428.**, 402.01, 402.11, 402.91, 398.91, 404.01, 404.03, 404.11, 404.13, 404.91, 404.93 | I50.**, I11.0, I09.81, I13.0, I13.2 |  |

CVD: cardiovascular disease; ICD-9: International classification of diseases 9^th^ revision; ICD-10: International classification of diseases 10^th^ revision; CPT4: current procedure terminology, 4^th^ edition
