# Supplementary material for: High-sensitivity troponin I is associated with cardiovascular outcomes but not with breast arterial calcification among postmenopausal women
Source: Int J Cardiol Cardiovasc Risk Prev. 2022 Nov 1;15:200157. doi: 10.1016/j.ijcrp.2022.200157 (PMC9789357; doi:10.1016/j.ijcrp.2022.200157)
Supplement: Multimedia component 3 [file mmc3.docx]

**Table S2.** Calibration, Discrimination and Reclassification of ASCVD (CHD + ischemic stroke; n=2,896; 81 events).

| Variables in the Model |  |
| --- | --- |
| Calibration  (Greenwood-D'Agostino-Nam Test)  Chi-square (df); p-value | |
| PCE only | 8.0 (df=9); 0.53 |
| PCE + Hs Tn I continuous | 8.3 (df=9); 0.51 |
| PCE + Hs Tn I categorical* | 16.9 (df=9); 0.05 |
| Discrimination  C-index (95% CI); p-value | |
| PCE only | 63.4 (59.3, 67.4) |
| PCE + Hs Tn I continuous | 64.3 (60.3, 68.5)  0.28 |
| PCE + + Hs Tn I categorical* | 64.0 (60.0, 68.0)  0.27 |
| Overall Category-based† Net Reclassification Improvement [NRI] (95% CI); p-value | |
| Adding Hs Tn I continuous | 0.12 (0.03,0.22); 0.01 |
| Adding Hs Tn I categorical* | 0.07 (0.00,0.14); 0.06 |
| Bias-corrected clinical Net Reclassification Improvement [cNRI] (95% CI); p-value | |
| Adding Hs Tn I continuous | 0.11 (0.01,0.22); 0.04 |
| Adding Hs Tn I categorical* | 0.07 (0.00,0.15); 0.07 |

Hs Tn I: high-sensitivity troponin I; PCE: pooled cohorts equation

* 3-categories of Hs Tn I: < 4, 4-10, >10 ng/L

PCE was transformed using a log(-log(1-x)) function

Categories of PCE: <5%, 5 to <7.5%, 7.5 to <20%, ≥ 20%
