# Supplementary material for: High-sensitivity troponin I is associated with cardiovascular outcomes but not with breast arterial calcification among postmenopausal women
Source: Int J Cardiol Cardiovasc Risk Prev. 2022 Nov 1;15:200157. doi: 10.1016/j.ijcrp.2022.200157 (PMC9789357; doi:10.1016/j.ijcrp.2022.200157)
Supplement: Multimedia component 4 [file mmc4.docx]

**STable 3.**

**NRI reclassification table for expected ASCVD (CHD + ischemic stroke) events by Kaplan-Meier** **using 5-year time horizon**

| Model with PCE only | Model with PCE + Hs Tn I continuous | | | |
| --- | --- | --- | --- | --- |
|  | <5% | 5 to < 7.5% | 7.5 to < 20% | >=20% |
| <5% | 13 | 4 | 1 | 0 |
| 5 to < 7.5% | 2 | 17 | 6 | 0 |
| 7.5 to < 20% | 0 | 4 | 47 | 7 |
| >=20% | 0 | 0 | 3 | 7 |

**NRI reclassification table for expected non-ASCVD events by Kaplan-Meier using 5-year time horizon**

| Model with PCE only | Model with PCE + Hs Tn I continuous | | | |
| --- | --- | --- | --- | --- |
|  | <5% | 5 to < 7.5% | 7.5 to < 20% | >=20% |
| <5% | 1,446 | 187 | 34 | 0 |
| 5 to < 7.5% | 372 | 460 | 265 | 0 |
| 7.5 to < 20% | 0 | 357 | 1,310 | 157 |
| >=20% | 0 | 0 | 107 | 246 |

NRI + = 0.084; NRI - = 0.039; NRI = 0.123

Crude clinical NRI = 0.189; Expected clinical NRI = 0.074; corrected clinical NRI = 0.115

ASCVD: atherosclerotic cardiovascular disease; Hs Tn I: high-sensitivity troponin I; NRI: net reclassification improvement; PCE: pooled cohorts equation

**NRI reclassification table for expected ASCVD (CHD + ischemic stroke) events by Kaplan-Meier using 5-year time horizon**

| Model with PCE only | Model with PCE + Hs Tn I categorical* | | | |
| --- | --- | --- | --- | --- |
|  | <5% | 5 to < 7.5% | 7.5 to < 20% | >=20% |
| <5% | 13 | 4 | 1 | 0 |
| 5 to < 7.5% | 2 | 17 | 6 | 0 |
| 7.5 to < 20% | 0 | 4 | 47 | 7 |
| >=20% | 0 | 0 | 3 | 7 |

**NRI reclassification table for expected non-ASCVD events by Kaplan-Meier using 5-year time horizon**

| Model with PCE only | Model with PCE + Hs Tn I categorical* | | | |
| --- | --- | --- | --- | --- |
|  | <5% | 5 to < 7.5% | 7.5 to < 20% | >=20% |
| <5% | 1,446 | 187 | 34 | 0 |
| 5 to < 7.5% | 372 | 460 | 265 | 0 |
| 7.5 to < 20% | 0 | 357 | 1,310 | 157 |
| >=20% | 0 | 0 | 107 | 246 |

NRI + = 0.084; NRI - = 0.039; NRI = 0.123

Crude clinical NRI = 0.189; Expected clinical NRI = 0.074; corrected clinical NRI = 0.115

ASCVD: atherosclerotic cardiovascular disease; Hs Tn I: high-sensitivity troponin I; NRI: net reclassification improvement; PCE: pooled cohorts equation; * 3-categories of Hs Tn I: < 4, 4-10, >10 ng/L
