# Supplementary material for: High-sensitivity troponin I is associated with cardiovascular outcomes but not with breast arterial calcification among postmenopausal women
Source: Int J Cardiol Cardiovasc Risk Prev. 2022 Nov 1;15:200157. doi: 10.1016/j.ijcrp.2022.200157 (PMC9789357; doi:10.1016/j.ijcrp.2022.200157)
Supplement: Multimedia component 5 [file mmc5.docx]

**STable 4.** Incidence of Failure Types (Age-adjusted Rates per 1,000 Person-years and SE) by hs TnI levels (n=2,896).

| hs TnI (ng/L) | Heart Failure with Preserved* Ejection Fraction  (n=29) | Heart Failure with Reduced† Ejection Fraction  (n=17) |
| --- | --- | --- |
| < 4  n=1,715 (59.2%) | n=14  1.1 ± 2.7 | n=2  0.1 ± 3.6 |
| 4 – 10  n=1,039 (35.8%) | n=14  1.7 ± 2.7 | n=7  0.6 ± 3.6 |
| >10  n=142 (4.9%) | n=1  0.9 ± 2.9 | n=8  5.1 ± 3.6 |
| p-trend | 0.61 | <.0001 |

*: EF ≥ 50%; †: EF < 50%; The CVD events are through Sept 30, 2021.
