# Supplementary material for: High-sensitivity troponin I is associated with cardiovascular outcomes but not with breast arterial calcification among postmenopausal women
Source: Int J Cardiol Cardiovasc Risk Prev. 2022 Nov 1;15:200157. doi: 10.1016/j.ijcrp.2022.200157 (PMC9789357; doi:10.1016/j.ijcrp.2022.200157)
Supplement: Multimedia component 6 [file mmc6.docx]

| hs TnI (ng/L) | Heart Failure with Preserved Ejection Fraction  (n=29) | Heart Failure with Reduced Ejection Fraction  (n=17) |
| --- | --- | --- |
| < 4 (n=1,715; 59.2%)  Model 1  Model 2 | 1.00  1.00 | 1.00  1.00 |
| 4 – 10 (n=1,039; 35.8%)    Model 1    Model 2 | 1.60 (0.76 – 3.38); 0.22  1.37 (0.64 – 2.90); 0.42 | 4.75 (0.98 – 22.99); 0.05  4.08 (0.83 – 20.12);0.08 |
| >10 (n=142; 4.9%)  Model 1  Model 2 | 0.88 (0.12 – 6.70); 0.90  0.85 (0.11 – 6.48); 0.87 | 43.10 (9.10 – 203.98); <.0001  40.60 (8.38 – 196.75); <.0001 |

**STable 5.** Independent Associations of high-sensitivity Troponin I with Heart Failure Types (n=2,896).

CVD events are through Sept 30, 2021.

*: EF ≥ 50%; †: EF < 50%

Model 1: age- and race/ethnicity-adjusted

Model 2: adjusted for age, race/ethnicity, smoking, BMI, hypertension, glycemic status, total cholesterol/HDL ratio, cholesterol lowering drugs, hs-CRP.
